# Supplementary material for: Five millennia of Bartonella quintana bacteraemia
Source: PLoS One. 2020 Nov 4;15(11):e0239526. doi: 10.1371/journal.pone.0239526 (PMC7641340; doi:10.1371/journal.pone.0239526)
Supplement: Attachment — Submitted filename: Rebuttal Letter.docx [file pone.0239526.s003.docx]

**Dear Editor,**

**Please find enclosed the second revised version of the manuscript PONE-D-20-07460 entitled: “Five millennia of *Bartonella quintana* bacteremia” along with the answers of the authors to the reviewers ‘ comments.**

Reviewers' comments:

Reviewer #1: This new version of the manuscript is well-researched and presented in a better form. In my opinion, the authors have made good amendments.

The "La Sicilia" reference be may grey literature but I am very skeptical about going to the media with certain claims and then issuing something different in a scientific paper, but thanks for clarifying that.

**Authors’answer: The authors acknowledge this positive remark.**

I would only recommend adding a couple of general references when you discuss the pathogen-population interaction:

1) Bos KI, Kühnert D, Herbig A, et al. Paleomicrobiology: Diagnosis and Evolution of Ancient Pathogens. Annu Rev Microbiol. 2019;73:639-666.

2) Rühli FJ, Galassi FM, Haeusler M. Palaeopathology: Current challenges and medical impact. Clin Anat. 2016;29(7):816-822.

**Authors’answer: The reviewer is perfectly suggesting to broaden the Discussion section, these two references are now cited in the Discussion section (Lines 213-215), and listed as new references 44 and 45, accordingly.**

Reviewer #2: Edits made in an orderly and clear way. I think the authors have answered the main critical points of the previous version of this manuscript.

**Authors’answer: The authors acknowledge this positive remark.**

**As the authors answered the Reviewer n°1 remark and corrected the manuscript accordingly, the authors hope that this second revised version will be accepted for publication,**

**Sincerely**

**Gérard ABOUDHARAM,**

**Corresponding author.**
